# Influence of green technology, green energy consumption, energy efficiency, trade, economic development and FDI on climate change in South Asia

**DOI:** 10.1038/s41598-022-20432-z

**Published:** 2022-09-30

**Authors:** Gulzara Tariq, Huaping Sun, Imad Ali, Amjad Ali Pasha, Muhammad Sohail Khan, Mustafa Mutiur Rahman, Abdullah Mohamed, Qasim Shah

**Affiliations:** 1 School of Finance & Economics, Jiangsu University, Zhenjiang, 212013 Jiangsu China; 2 School of Economics and Management, China University of Geosciences, 430078, Wuhan, China; 3 Aerospace Engineering Department, Faculty of Engineering, King Abdulaziz University, Jeddah 21589, Saudi Arabia; 4 School of Mathematical Sciences, Jiangsu University, Zhenjiang, 212013 Jiangsu China; 5 Department of Mechanical and Mechatronics Engineering, University of Waterloo, Waterloo, ON N2L 3G1 Canada; 6 Research Centre, Future University in Egypt, New Cairo, 11835 Egypt; 7 Department of Statistics, University of Peshawar, Peshawar, Pakistan

**Keywords:** Ecology, Environmental sciences

## Abstract

Climate change policy has several potential risks. The purpose of this study is to investigate the impact of green technology development, green energy consumption, energy efficiency, foreign direct investment, economic growth, and trade (imports and exports) on greenhouse gas (GHG) emissions in South Asia from 1981 to 2018. We employed Breusch Pagan LM, bias-corrected scaled LM, and Pesaran CD as part of a series of techniques that can assist in resolving the problem of cross-sectional dependence. First and second generation unit root tests are used to assess the stationarity of the series, Pedroni and Kao tests are used to test co-integration. The long-term associations are examined using fully modified ordinary least square (FMOLS) and panel dynamic ordinary least square (DOLS) for robustness. The results revealed that trade, growth rate, and exports significantly increase GHG emissions. This accepted the leakage phenomenon. The results also demonstrated that green technology development, green energy consumption, energy efficiency, and imports all have a significant negative correlation with GHG emissions. Imports, advanced technical processes, a transition from non-green energy to green energy consumption, and energy efficiency are thus critical components in executing climate change legislation. These findings highlight the profound importance of green technology development and green energy for ecologically sustainable development in the South Asian countries and act as a crucial resource for other nations throughout the world when it comes to ecological security. This research recommends the consumption of environmentally friendly and energy-efficient technologies in order to mitigate climate change and the government's implementation of the most recent policies to neutralize GHG emissions in order to achieve sustainable development.

## Introduction

One of the world's most serious issues is climate change. In almost all countries, attempts are being made, irrespective of economic expansion level, to slow the rate of greenhouse gas emissions (GHG)^1^. Recent conferences on emissions reduction have encouraged more sustainable behavior, actively managing potential effects on the environment and local community, and promoting resource management throughout the supply chain^2^. Dong et al.^3^ investigated the influence of economic growth on world panel GHG emissions. When evaluating the effects of economic development on the environment, various scholars emphasized the distinct impacts of economic progress on GHG emissions at various stages of development, which corresponds to the Environmental Kuznets Curve (EKC) theory^4,5^. According to EKC, economic growth and the environment have an inverted U-shaped connection. EKC is the degree of pollution that rises as countries grow but begins to fall once income rises over a certain threshold^6–10^.

Furthermore, increased per capita economic development will not reduce GHG emissions^11,12^. Economic expansion has an impact on the environment as a result of scale, composition, and technology impacts^13^. The scale effect illustrates that more energy consumption and production emit more pollutants than the quantity of GHG emissions rises. When growth in the economy promotes a structural change in the economy toward less polluting activity, the composition effect occurs. Technique effect states that developed economies use more resources for the replacement of dirty technology with clean technology when the quality of the environment is improved^14^. So, these assumptions state that there are other factors except GDP that negatively and positively affect the EKC hypothesis.

Various studies have determined that green or renewable energy resources have a significant role in reducing GHG emissions owing to clean energy sources and technical advancement^15–17^. FDI promotes improved technology, resulting in a cleaner environment^18,19^. However, the key issues with climate change policy are the globalization and expansion of the economy, which enhance output with the usage of energy, causing a rise in GHG emissions^20–23^. Furthermore, when assessing the influence of trade on GHG emissions, it is critical to account for leakage, which has been overlooked in prior studies. The leakage phenomenon is the process by which GHG emissions fall as industry transfers from developed to developing nations^24,25^. As a result of stricter rules, wealthy nations relocate their polluting sectors to poorer countries, thereby reducing their own emissions.

However, the literature has not taken into account the effects of green technology, green energy consumption, energy efficiency, trade, economic development, and FDI on climate change in South Asia. In order to close this research gap, this analysis increases the relationship between green technology, green energy consumption, energy efficiency, trade, economic development, FDI, and climate change. Therefore, the primary goals of climate change policy must be to increase green energy consumption, energy efficiency, and green technology development in order to slow down the growth rate of GHG emissions. Thus, the primary goal of this research is to assess the major factors that influence GHG emissions, both adversely and favorably. This study will give more precise insights into how to properly execute climate change policies in South Asian countries.

A few things about this research set it apart from earlier studies. To the best of our knowledge, this is the first research that examines the special effects of green technology development, green energy consumption, energy efficiency, trade, economic development, and FDI on climate change in the South Asian economies between 1981 and 2018. The study's findings will enable economists to better understand the environmental effects of green technology, green energy consumption, and energy efficiency, which will aid in the development of appropriate environmental policies. They will also help better predict the importance of green technology and green energy consumption in the reduction of ecological pollution. Second, this is the first research to examine the effects of green technology, green energy consumption, energy efficiency, trade, economic development, and FDI on GHG emissions, which have been ignored in other studies. Third, in order to address problems like endogeneity, heteroscedasticity, cross-sectional dependence, autocorrelation, and the presence of regression coefficients with different integration levels, this study uses robust estimators like panel dynamic ordinary least squares (DOLS) and panel fully modified ordinary least squares (FMOLS).

The following is how the research work is structured: The related literature is in point 2. Data and methodology are in point 3. Empirical findings and discussion are in point 4 and the conclusion & policy suggestions are delivered in point 5.

## Previous literature

According to the majority of studies, economic development is a primary factor in GHG emissions ^26–31^. Hence, the EKC theory indicates that environmental degradation grows higher when economic growth per capita increases until the turning point, then the reduction in environmental degradation is detected.

Natural resource rents were used in BRICS nations to investigate the connection amongst carbon emissions, economic development, renewable energy usage, and natural resources^32^. Using total natural resource rents, Balsalobre-Lorente et al.^33^ evaluated the link between renewable power, economic progress, natural resources, and carbon dioxide emissions. Numerous researchers have attempted to study the relationship between the use of renewable energy, economic growth, and ecological damage^33–36^.

According to Omri et al.^37^, FDI contributes to environmental degradation in Saudi Arabia, whereas^38^ discovered a decline in environmental damage in Asian nations as a result of FDI. According to^39^, FDI has an important influence on environmental deterioration and investment in sustainable energy lowers environmental devastation. Zhang and Zhou^40^ investigated the association of FDI and environmental deterioration in China and concluded that FDI lowers environmental damage and the equipment level and industrial constitution reduce environmental pollution in China. Phung et al.^41^ supports the pollution halo hypothesis that claims that foreign direct investment may stimulate green growth in south-east Asian nations. Quang et al.^42^ explained how green bonds have a short-term detrimental effect on energy intensity. In addition, factors that might influence energy intensity include per capita income, economic integration, and the availability of renewable energy sources, whereas ASEAN modernization could influence energy intensity in the other direction.

The 4th Industrial Revolution is about to hit the world economies, and technological innovation is thought to be the main mechanism for achieving sustainable development. In this respect, it is reasonable to anticipate that technology advancements will affect environmental elements as well ^43,44^. More significantly, eco-innovation is thought to be the solution to the world'senvironmental problems^45–47^. Similar to this, Ding et al.^48^ cited eco-innovation as being in responsible of lowering CO_2_ emissions levels in the G7 nations. Numerous academics have also published similar results, including Zhang et al.^49^ for China, for the United States Solarin and Bello^50^, Hashmi and Alam^51^ for OECD nations, and Sinha et al.^52^ for the N11 economies. Similar to this, Gormus and Aydin^53^ discovered that technological advancements lowering long-term pollution levels in OECD countries. Additionally, the country-specific findings demonstrate that technology advancements are effective in limiting environmental pollution in the US, Finland, and Korea, and the US. Usman and Hammar^54^, on the other hand, concluded that advanced technologies deteriorate the environmental quality in the APEC countries. As a result, it might be claimed that the effects of technological advancements on the environment are sometimes unclear.

Trade is one of the main factors which improve growth^55,56^. Moreover, trade consists of two variables as exports and imports. The majority of researchers measured the openness of trade by exports plus imports to GDP ratio^57,58^. In the EKC theory this proxy is not valid as it involves two variables but it is valuable to include both because exports are directly related to increasing the level of production but imports reduce the production level. Numerous studies considered import and export instead of trade openness^59,60^. According to Jebli and Youssef^61^, imports and exports per capita in Tunisia had positive relationship with GHG emissions per capita. Furthermore, Jebli et al.^62^ demonstrated imports and exports in OECD nations minimize GHG emissions. In the case of UAE increase in exports reduced the GHG emission in long-term^63^. Xu and Lin^64^ revealed that exports reduce the GHG emission in China. Additionally, the study's inclusion of both exports and imports data might reveal the leakage phenomena. Leakage phenomenon is transferring dirty industries from developed to underdeveloped countries through trade^65,66^. Meanwhile, in underdeveloped countries growth of export and production could cause an increase in GHG emission.

Energy efficiency and green energy are predicted to be the major indicators in climate change policy that can alleviate the problem of climate change. Different scholars^67–70^ demonstrated energy efficiency can help to minimize the rise of GHG emissions. Özbuğday and Erbas^71^ discovered that 24 of 36 countries' energy-efficiency strategies are efficient at lowering GHG emissions. Energy intensity is a negative factor in slowing the pace of growth of GHG emissions^72–75^. Sharif et al.^76^ established a bidirectional relationship among GHG emissions and energy intensity in Turkey. Green energy is an alternate to carbon energy that actively helps in reduction of GHG emissions. According to Khan et al.^77^, GHG emissions in the United States are adversely associated to renewable energy. The similar conclusion was verified in 17 OECD countries^14^. Renewable energy usage in EU countries, Bölük and Mert^78^ indicated that it may reduce energy consumption by 50% compared to fossil energy. Underdeveloped nations such as Tunisia, Indonesia, Malaysia, Turkey, India, and the BRICS have stated that renewable energy usage has a negative influence on GHG emissions^79–82^. Apergis and Payne^83^ discovered that renewable energy usage had no effect on GHG emissions in a panel of 19 developing and developed nations. For the instance of the United States, Menyah and Wolde-Rufael^84^ revealed unidirectional causation between renewable energy usage and GHG emissions^85^. concluded that economic growth and the use of renewable energy have a positive and negative impact on environmental pollution, respectively. Innovation activity considerably and negatively moderates the association between financial inclusion and environmental degradation in all quantile distributions. In the long term, Adebayo^86^ found that the usage of renewable energy, political risk, and trade globalization all help to slow down environmental degradation.

FDI is an indicator that enhances economic growth through the introduction of managerial skills, production processes, productivity gains, and technology transfer^87,88^. Foreign investors use advanced technology and management skills to clean up the environment of host countries. So, FDI promotes technology innovation, low carbon growth, and increases energy efficiency^89,90^. The Pollution Haven Hypothesis states that FDI supports economic growth at the expense of the environment. Adebayo^91^ analyzed the relationship between variables at various frequencies and different timeframes using the innovative wavelet coherence approach. The results of the wavelet correlation showed that renewable energy consumption improves environmental quality in the short and medium term; fossil fuel use degrades environmental quality in the short and medium term; FDI inflows improve environmental quality at all frequencies; and economic complexity impairs environmental quality in the short, medium, and long term. Adebayo et al.^92^ indicate that at various quantiles, environmental degradation is accelerated by globalization, tourism, economic expansion, and energy consumption. Akadiri et al.^93^ concluded a positive relationship between ecological footprint and the usage of renewable and non-renewable energy, economic growth, and economic complexity. Using ARDL, Xie et al.^94^ showed that increases in economic growth and structural change result in increases in CO_2_, while increases in renewable energy and technological innovation result in decreases in CO_2_ emissions. Du et al.^95^ disclosed that high-tech industry, economic growth, and FDI increase CO_2_ emissions, while renewable energy consumption alleviates CO_2_ emissions.

Moreover, In the Gulf Corporation Council, Al-Mulali and Tang^96^ discovered that FDI had a neutral influence on GHG emissions. FDI increases GHG emissions in China, Malaysia, and Sub-Saharan Africa^97,98^. Although, FDI lower the GHG emissions in developing countries like Vietnam and China^99^. Omri and Kahouli^100^ focused on the three regions: Latin America and the Caribbean, Europe and Central Asia, North and Sub-Saharan Africa, and the Middle East, and discovered bidirectional causation among GHG emissions and FDI with the exception of North Asia and Europe. However, research on the effects of energy efficiency, green energy use, green technology development, economic development, trade and FDI on GHG emissions in South Asian countries is lacking. As a result, the purpose of this research is to fill that void.

## Material and methods

### Theoretical analysis

Green technology is used as a green source to lower emissions. Use of green technology has led to a decrease in the trend of environmental deterioration. Moreover, in recent decades, nations have changed the way they consume energy to promote green sources. However, these energies have a significant effect on environmental degradation. Regardless of whether all economies use green energy and green technology, therefore, it is crucial to understand why this issue has not yet been solved. As a result, this study evaluated the relationship between green technology development and environmental pollution. Model may be expressed as;

1 $$GHG = f(GT,GE,EF)$$

where GT is green technology development, the development of environment-related technology is used as a proxy for green technology.

Dong et al. ^101^ specified that green energy burns more cleanly than petroleum and coal. To further evaluate the impacts of green energy consumption, green technology development, FDI, trade, and energy efficiency on GHG emission in South Asia, this study extended the Eq. (1) as follows:

2 $$GHG = a_{0} + \delta_{1} GDP + \delta_{2} GE + \delta_{3} EF + \delta_{4} FDI + \delta_{5} IMP + \delta_{6} EXP + \delta_{7} GT$$

where α is intercept, GE is green energy, where renewable energy is utilized as a proxy of green energy, EF refers to energy efficiency, FDI is foreign direct investment inwards, IMP represents imports, EXP denotes exports, and GT is green technology development.

All variables are transformed to natural logarithms before the model is estimated in order to standardize the data and produce accurate estimates by supporting the evaluation of the regression coefficient elasticity. As a result, Eq. (2)'s panel log-linear econometric functions may now be written as:

3 $$lGHG_{it} = \alpha_{0} + \delta_{1} lGDP_{it} + \delta_{2} lGE_{it} + \delta_{3} lEF_{it} + \delta_{4} lFDI_{it} + \delta_{5} lIMP_{it} + \delta_{6} lEXP_{it} + \delta_{7} lGT_{it} + \varepsilon_{it}$$

where *i* signifies number of countries, t indicates time period and *ε* is the error term. $\delta_{1}$–$\delta_{7}$ refer coefficients of variables.

### Empirical data

**Figure 1:**
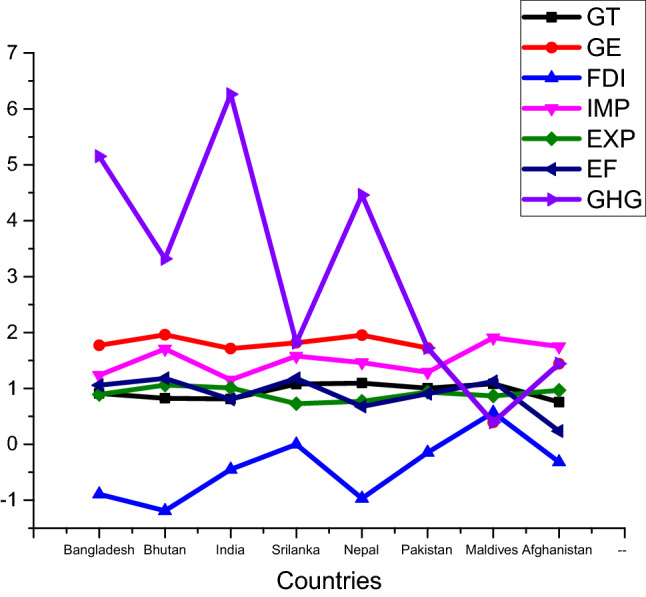
Data Distribution.

The source of data is described in Table 1. This research employs panel data for all selected variables except green technology development, which is collected from WDI^102^ o meet the objectives. The eight data series include GHG emissions (GHG), green technology development (GT), energy efficiency (EF), renewable energy consumption (REC), which is used as a proxy for green energy (GE), imports (IMP), exports (EXP), and foreign direct investment (FDI), gross domestic product (GDP) are used in this research. The time period of this study is 1981–2018. A graphical representation of data is presented in Fig. 1. Figure 1 showing GHG emissions in all countries are very high. A description of the variables is provided in Table 2. According to available data, averages are quantified as GHG (11.93), GDP (25.147), FDI (−0.95), GE (3.647), EF (2.176), IMP (3.432), and EXP (2.061). It is presented in Table 2 that all of the variables except green technology are negatively skewed.

**Table 1:** Description of Variables.

Variable name	Short variable name	Measurement unit	Database
Green technology development	GT	Development of Environment related Technologies, % of all technologies	OECD (2021)^103^
Greenhouse gas	GHG	Kilo tons	WDI^102^
Foreign direct investment	FDI	Foreign direct investment, net inflows (% of GDP)	WDI^102^
Imports	IMP	Imports of goods and services (% of GDP)	WDI^102^
Exports	EXP	Exports of goods and services (annual % growth)	WDI^102^
Gross domestic product	GDP	PPP (constant 2011 international $)	WDI^102^
Energy efficiency	EF	GDP per unit of energy use (constant 2011 PPP $ per kg of oil equivalent)	WDI^102^
Green energy	GE	Renewable energy consumption (% of total final energy consumption)	WDI^102^

**Table 2:** Descriptive Statistics.

	lGHG	lFDI	lGT	lGDP	lGE	lEF	lIMP	lEXP
Mean	11.93891	− 0.956198	2.124657	25.14782	3.647755	2.176103	3.432587	2.061388
Median	11.93944	− 0.406377	2.082339	25.26813	4.051794	2.160802	3.443213	2.241505
Maximum	14.91509	2.725624	3.703275	29.78354	4.563514	3.163486	5.342406	4.449841
Minimum	7.388862	− 12.50914	0.029559	20.94177	− 0.101997	1.355783	1.954160	− 3.557694
Std. Dev	1.852061	2.470256	0.769758	2.325420	1.165537	0.439537	0.644668	1.084517
Skewness	− 0.158254	− 2.479081	0.131183	− 0.155402	− 1.893202	− 0.041275	− 0.011775	− 1.365102
Kurtosis	2.627072	11.55802	2.142407	2.190686	5.437573	2.110700	2.660007	7.601079

Beginning with the baseline model, the Pesaran CD, Pesaran scaled LM, and Breusch-Pagan LM tests were used to examine the cross-sectional dependency of variables. The following stage is to look at variable stationarity in panel data with cross-sectional dependence. Each panel unit root test has advantages and disadvantages^57^. In this work, three-unit root tests are used to increase robustness: Levin Lin and Chu^104^, Breitun^105^, and lm Pesaran and Shin^106^. The panel unit root test, devised by Levin Lin and Chu^104^ is an expansion of the enhanced Dickey-Fuller test, which is stated as:

4 $$\Delta D_{it} = \delta_{it} \psi_{i} + hD_{it - 1} + \sum\limits_{p = 1}^{mi} {\delta ik} D_{i,t - p} + \xi_{it}$$

where *h* represents autoregressive coefficients, $\delta_{it}$ indicated individual deterministic variable, m is lag order, and $\xi$ representing error term.

It is assumed in the Levin, Lin, and Chu^104^ test that h remains constant across countries. Levin, Lin, and Chu^104^ the test is prolonged from Im, Pesaran, and Shin^106^ test, which lets h to vary among countries. Breitung^105^ is a test that corrects for bias produced by the LLC^104^ and IPS^106^ tests, as well as supplied given equation:

5 $$D_{it} = \phi_{it} + \sum\limits_{j = 1}^{h + 1} {D_{ij} a_{it - t} } + \varepsilon_{it}$$

According to Hlouskova and Wagner, and Narayan and Narayan^107,108^, the IPS^105^ test has some limitations and advantages too. The best benefit is that it has the highest power and the smallest sample size biases, whereas the limitation is that the autoregressive coefficient remains the same across countries.

Pedroni^109,110^ developed panel and group co-integration tests. Panel rho-Statistics, Panel v-Statistics, Panel pp-Statistics, and Panel ADF-Statistics are all within dimension approaches used by the panel test. It also contains between dimension approaches: Group ADF-Statistics, Group PP-Statistics, and Group rho-Statistics. Pedroni's cointegration assessment^109,110^ assumes that H0: no co-integration among variables. These seven tests, which are asymptotically dispersed as regular standards, are defined as the expected residuals from the long-run model shown below.

6 $${\text{D}}_{{{\text{it}}}} { = }\phi_{{\text{i}}} { + }\lambda_{i} { + }\sum\limits_{{\text{k = 1}}}^{{\text{n}}} {\beta_{{{\text{ik}}}} C_{{{\text{kit}}}} } { + }\varepsilon_{{{\text{it}}}}$$

where C and D are planned to be incorporated into order one levels.

In Eq. (6) the projected residuals are recorded.

7 $$\varepsilon_{{{\text{it}}}} = h_{i} \varepsilon_{{\text{it - 1}}} + \mu_{it}$$

The maximum likelihood-based panel co-integration statistics will be compared to three between-dimension and four within-dimension statistics in this study.

Pedroni's study of the cointegration system for panel data is stated in Eq. (8) below:

8 $$D_{{{\text{it}}}} = \phi_{i} + \beta C_{it} + \varepsilon_{it}$$

Kao^111^ proposes another co-integration test to estimate the homogeneous co-integration association. Kao proposes two tests for the null hypothesis of no co-integration: the Dickey-Fuller type and the Augmented Dickey-Fuller tests.

After establishing panel co-integration, this study examines the long run relationship between variables using panel fully modified ordinary least squares (FMOLS) and panel dynamic ordinary least squares (DOLS) to avoid the problem of serial correlation between GHG emissions, FDI, energy efficiency, trade, economic growth, green technology development, and green energy consumption. It is expected that green energy consumption, green technology development, and energy efficiency have a negative link with GHG emissions. Therefore,$\delta_{2}$, $\delta_{3}$ and $\delta_{7}$ < 0.

## Results and discussions

### Cross-sectional dependence test

To avoid biased and inconsistent estimations, cross-section dependency tests are performed prior to the stationary test. To examine cross-sectional dependency, three approaches are used: the Breusch-Pagan Lagrange multiplier (LM) test^112^, the Pesaran scaled LM test^113^, and the Pesaran cross-section dependence (CD) test^114^. Table 3 displays the results. As the probability is < 0.5, the null hypothesis is rejected as H0: Variables do not have a serial correlation.

**Table 3:** Baseline Model.

Variables	Coef.	t-test	Pr.
GE	− 0.711455	− 2.809721	0.0061
EF	− 0.778755	0.109876	0.0000
GT	0.004796	4.391927	0.8288
FDI	− 0.058448	− 2.388415	0.0190
GDP	0.828632	20.72483	0.0000
IMP	− 0.478040	− 5.279160	0.0000
EXP	0.066690	3.020474	0.0033
C	− 4.045237	− 1.879908	0.0634

Serial correlation			
Test	Stat.	Pr.	
Breusch-Pagan LM	15.72042	0.0013	
Pesaran scaled LM	5.193090	0.0000	
Pesaran CD	3.832597	0.0001	

### Second generation unit root test

Levin Lin and Chu^104^, Im Pesaran and Shin^106^, and Breitun^105^ unit root test are accessible in Table 4. Table 4 revealed the probability of FDI and green technology development is less than 5% as they are I(0) so there is no unit root except GHG, GT, GDP, GE, and EF so they are I(1).

**Table 4:** Unit Root Test.

Vr	LLC		Breitung		CIPS	
	Level	1st diff.	Level	1st diff.	Level	1st diff
lGHG	− 1.16781	− 3.15327***	0.26418	− 5.73555***	2.04830	− 8.68736***
lGT	− 1.89452**	− 7.78981***	− 2.26184**	− 3.82286***	− 0.67430	− 5.67483***
lFDI	− 1.39536**	− 10.5733***	− 4.22134***	− 4.49438***	− 4.76324***	− 12.6738***
lGDP	3.62628	− 5.65180***	0.80827	− 7.36270***	1.31810	− 5.69569***
lIMP	0.30058	− 7.6.835***	− 0.70295	− 7.23184***	− 0.87331	− 8.09093***
lEXP	− 0.20032	− 2.01365**	1.15181	− 2.50794***	− 0.32172	− 3.99091***
lGE	0.42641	− 4.41745***	0.50730	− 6.74013***	0.40893	− 5.90183***
lEF	0.05227	− 6.35528***	1.16734	− 3.71679***	0.86602	− 7.12394***

Notes: The superscripts ***, ** & * signify statistical significant at the 1%, 5% and 10% respectively.

### Cointegration checks

To analyze the co-integration of variables, this study used two co-integration tests developed by Pedroni ^109,110^ and kao^111^. Table 5 summarizes the results of the co-integration tests. This study observed two heterogonous and three homogenous statistics of Pedroni and Kao^109,110^ are statistically significant at 1% in South Asian countries; This indicates that the alternative theory of co-existence integration's is recognized.

**Table 5:** Cointegration Test.

Residual cointegration test by Pedroni ^109,110^		
	Stat.	Pr.
Panel v-Statistics	5.668768	0.0000
Panel rho-Statistics	− 0.491786	0.3114
Panel pp-Statistics	− 1.402812	0.0803
Panel ADF-Statistics	− 5.013338	0.0000
Group rho-Statistics	2.771068	0.9972
Group PP-Statistics	− 5.819535	0.0000
Group ADF-Statistics	− 1.904962	0.0284
**Kao **^111^** cointegration test**		
ADF	− 1.391895	0.0820

### Long-run relationships

The co-integrating connections among the chosen variables are validated by the stationary test section. Yet, long-run coefficients of regressors including economic development, green energy usage, and development of green technology, FDI, and trade are not calculated. As a result, panel fully modified ordinary least squares (FMOLS) and panel dynamic ordinary least squares (DOLS) are used to compute the long-run coefficients. Stock and Watson, and Phillips and Hansen^115,116^ suggested FMOLS and DOLS, which were refined using panel data by Pedroni^117^ and Kao and Chiang^118^. Panel FMOLS and DOLS adjust for both simultaneous bias and serial correlation problem^115,117^.

Table 6 represents the outcomes of long-run estimates, which elaborated on the nexus among climate change (GHG emissions), green technology development (GT), energy efficiency (EF), green energy (GE), trade (IMP and EXP), economic growth (GDP), and foreign direct investment (FDI). DOLS research reveals that a 1% increase in FDI raises emissions by 31% while a 1% increase in imports lowers emissions by 2%. The result is in line with Zhang and Zhou, Liobikienė and Butkus, and Balsalobre-Lorente et al.^40,119,120^ results. The projected GDP result is positive and statistically significant at 9%, implying that a 1% increase in GDP increased GHG emissions by 2%.This confirms that the growth of GDP enhanced the emissions, which in turn polluted the environment. The result is consistent with Sarkodie and Adams^121^.

**Table 6:** Estimates using FMOLS and DOLS.

Vrs	FMOLS		DOLS	
	Coef	t-Stat.	Coef	t-Stat.
lGT	− 0.019684	− 1.185560	− 1.926096	− 4.838958*
lGDP	1.010009	27.8015***	0.026060	0.040715*
lFDI	0.102729	4.459799***	0.031209	2.582304***
lIMP	− 0.347588	− 4.049463***	− 0.020515	− 0.203986**
lEXP	0.009842	2.122036**	0.008959	− 2.401694**
lGE	− 0.825366	− 8.634595***	− 0.469519	1.038035
lEF	− 0.265219	− 0.735597	− 0.311651	1.807367**

Notes: The superscripts ***, ** & * signify statistical significant at the 1% , 5% and 10% respectively.

The relationship between exports and GHG emissions is positive and significant; a 1% increase in exports resulted in a 0.8 percent increase in emissions; this result is consistent with Mohamued et al. and Iqbal et al.^122,123^. Table 6 also reveals the negative and statistically significant relationship between energy efficiency and GHG emissions. The results showed that 1% increase in energy efficiency propelled the emissions by 31%. It is critical to remember that the use of renewable energy must be protracted, if the consumption is not sustainable then it might affect the environment^121^. So, in South Asia, green energy consumption has a negative nexus with the climate using the DOLS method.

Furthermore, Table 6 also elaborated on the results of FMOLS. In the long run, GDP is positive and significant at 1%. The evidence shows that a 1% increase in economic growth propels the environment by 101%, as confirmed by the results of Sarkodie and Adams^121^. Furthermore, the results also confirmed that the increase in FDI intensified the emissions by 10%. An increase in the share of imports lowered the emissions by 34%, through which environmental quality did not suffer. Export, in the long run, also enhances emissions at the expense of the environment by 7%. Hence, the connection between green energy consumption and GHG emissions is significantly negative; the results indicated that a 1% increase in green energy consumption minimized GHG emissions by 90%. In the long term, a percentage improvement in energy efficiency reduces GHG emissions.

**Figure 2:**
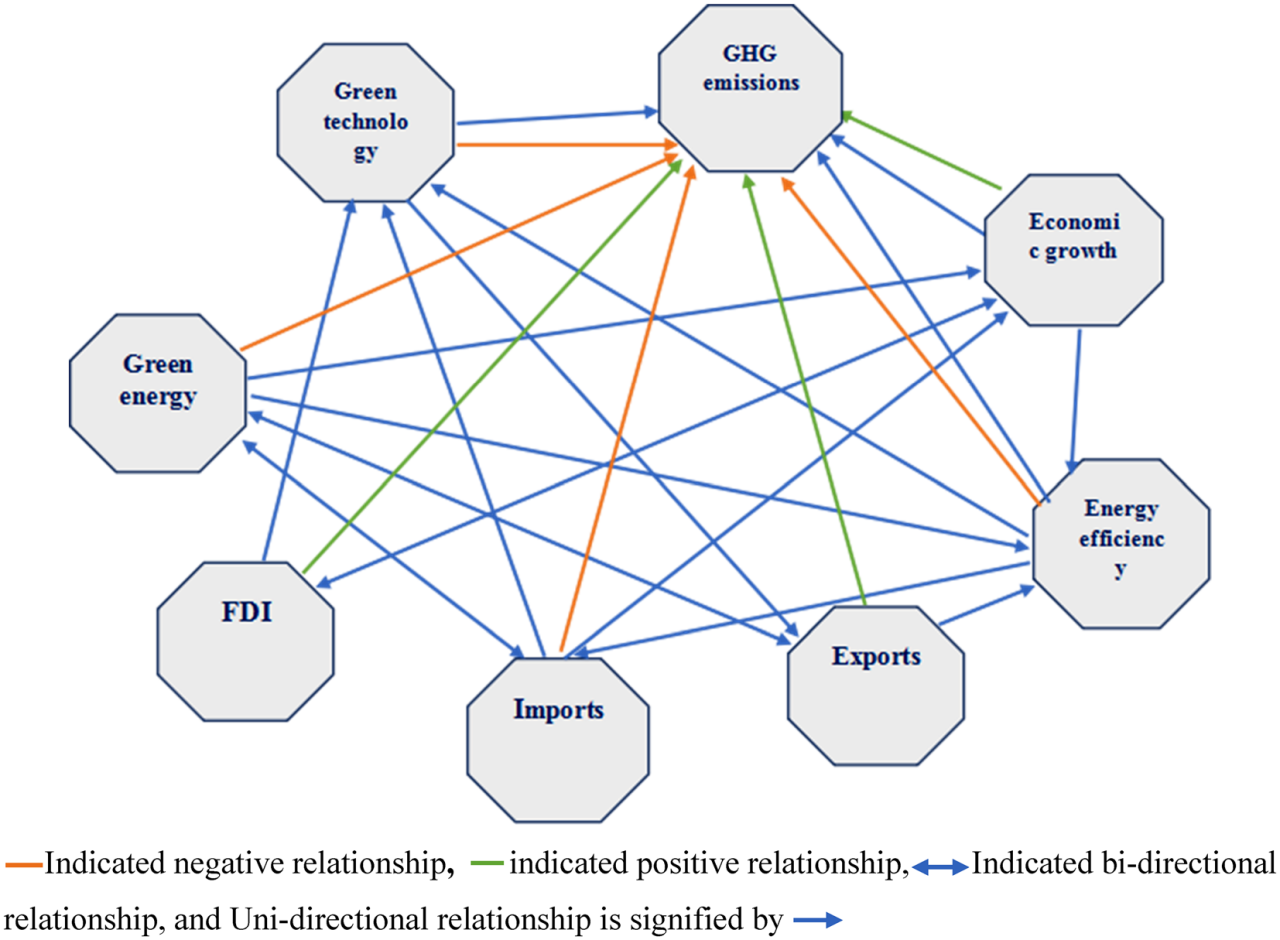
Geographical representation of results.

Moreover, in order to prevent biased or spurious results, different diagnostics were applied in this research in order to assess the independence of data. This study estimated the causality running from green technology, energy efficiency, green energy consumption, FDI, GDP, imports, and exports to climate change in Table 7. Table 7 reveals that there is bidirectional causality between imports, exports, and green energy. Moreover, there is also a bidirectional causality running from FDI to economic development. Green technology development, energy efficiency, economic development, and trade have unidirectional causality with climate change (shown in Fig. 2).

**Table 7:** Panel Causality Check.

Null Hypothesis	F-stat.	Null hypothesis	F-stat.
GT ⟹ GHG	4.12317**, 0.34254	EXP ⇔ GE	2.15361*, 2.10051*
EF ⟸ GHG	0.2897, 2.24250*	GDP ⟸ GE	1.10372, 2.18976*
GDP ⟹ GHG	2.52970**, 0.49555	IMP ⇔ GE	4.62725***, 3.20707**
EF ⟸ GT	0.24986, 3.86907**	EXP ⟹ EF	3.85201**, 0.35284
EXP ⟸ GT	0.07260, 2.91487**	GDP ⟹ EF	2.82000**, 0.11773
FDI ⟹ GT	3.94703**, 0.79086	IMP ⟸ EF	0.85024, 1.88561*
IMP ⟹ GT	1.73186*, 0.58104	FDI ⇔ GDP	2.17469*, 5.19241***
EF ⟸ GE	1.46212, 3.31186**	IMP ⟹ GDP	7.50591***, 1.06368

Note: The superscripts ***, ** & * signify statistical significant at the 1%, 5% and 10% respectively.

### Discussion

Climate change is one of the main problems in South Asian countries. Previous studies have not taken into account the effects of green technology, green energy consumption, energy efficiency, trade, economic development, and FDI on climate change in South Asia. In order to close this research gap, this analysis increases the relationship between green technology, green energy consumption, energy efficiency, trade, economic development, FDI, and climate change. Therefore, the primary goals of climate change policy must be to increase green energy consumption, energy efficiency, and green technology development in order to slow down the growth rate of GHG emissions. Thus, the primary goal of this research is to assess the major factors that influence GHG emissions, both adversely and favorably. This study will give more precise insights into how to properly execute climate change policies in South Asian countries.

The findings of an empirical investigation into the relationship between green energy consumption, energy efficiency, and trade, green technology development, economic growth, and GHG emissions reveal a lot about strategies and policies that can help to propel GHG emissions toward appropriate development goals. The commercial distribution of energy technologies comes with environmental compromises^124^. Hence, the effects of green energy consumption on long-term lower the GHG emissions. This study provided evidence that green energy consumption and energy efficiency reduced emissions while exports, GDP, and FDI accelerated climate change. Furthermore, adopting green energy not just improves environmental quality but also leads to energy-dependent economic reform, Sarkodie & Adams^121^. However, green energy sources cannot be traded compared to non-green energy sources, thus leading to economic productivity and energy security. Due to its numerous opportunities, green energy usage is directly related to long-term improvement, which is economic and social development, energy access, and reducing health and environmental impacts^125^.

Despite the fact that imports reduce production processes due to their contribution to GHG emissions, perhaps exports increase GHG emissions as they are associated with the increase in the production process. Also, an increase in exports increases economic growth and improved technologies could be utilized to lower emissions. Withdrawal of old technology, decontaminating strategies, and proper waste disposal could lower pollution as well as GHG emissions. In the countries under discussion, the ratio of green energy consumption is small as compared to non-green energy sources such as fossil fuels and coal, so aggregate consumption of energy accelerates GHG emissions. The IPCC reports reveal that energy consumption and its associated activities are the highest contributors to GHG emissions.

Climate change, economic changes, and policy are all rely heavily on FDI. Therefore, it is expected GHG emissions will decline with the use of green energy consumption, energy efficiency, and imports. Obviously, strong policies, institutions, leadership, and governance will decrease GHG emissions. Energy efficiency is demonstrated by lowering energy intensity while increasing economic productivity. Thus, FDI should be more directed to enhance innovation and new technologies that take part in the reduction of GHG emissions.

## Conclusion and policy implications

Climate change is a critical issue all around the planet. This study analyzed the impacts of the development of green technology, energy efficiency, green energy consumption, trade, FDI, and economic growth on GHG emissions. This study identified the most important climate change problems and possibilities for policymakers to consider. Furthermore, evaluating the factors of GHG emissions, economic development is significantly and positively inclined the GHG emissions. In South Asia, climate change remains the main challenge. While analyzing the impact of trade imports and exports, both variables are considered to assess the influence of separate variables. The results showed that GHG emissions decreased due to imports that accepted the leakage phenomenon.

Furthermore, economic growth increases GHG emissions in the long term. To solve the problem of climate change, countries should adopt the updated technological process to lower GHG emissions. Meanwhile, FDI significantly affects climate change. Thus, in South Asia, FDI has still not reached that stage where it significantly contributes to lowering GHG emissions. Moreover, increasing the share of green energy consumption, green technology development, and energy efficiency are the main opportunities to lower GHG emissions in South Asia. In South Asia, the growth rate of imports and a decrease in production should be witnessed, which might reduce GHG emissions. Regardless of the multiple obstacles, energy efficiency and the transition from non-green to green energy are the primary instruments for efficiently implementing climate change legislation.

There are several significant policy implications woven throughout the research findings. First, economic growth aggravates environmental pollution. The governments of South Asian nations have to adopt appropriate strategies to reduce their environmental pollution. South Asian governments must therefore implement the necessary measures to reduce climate change as economic expansion exacerbates it. Second, the negative impacts of green technology, green energy consumption, imports, and energy efficiency lead one to believe that South Asian countries could benefit from policies that encourage the development of green technology and use of green energy while simultaneously fostering economic growth. These regulations might include tax incentives for the development of green technology, as well as grants, subsidies, and refunds for the expansion of infrastructure. Moreover, green technology and renewable energy consumption have a negative and significant relationship with GHG emissions, which supports their reputation as green sources of energy with the ability to encourage ecological development and slow down the degree of environmental deprivation.

This study offers some innovative insights, but it also has significant limitations that may open up new areas for further study. The impacts of the development of green technology, energy efficiency, green energy consumption, trade, FDI, and economic growth on GHG emissions are contentious issues that are influenced by a variety of institutional, social, and cultural influences and are open to discussion. It also provided guidance for future research on other developing and emerging nations that consume non-green energy and produce more emissions, using both country-specific and panel data analysis to provide more precise information. Finally, expanding this research to include additional contributing aspects to various case studies, such as urbanization and natural resources, could produce compelling literature.

## Data Availability

The datasets used and/or analyzed during the current study available from the corresponding author on reasonable request.
